# Association Between Brain-Derived Neurotrophic Factor Val66Met Polymorphism and Methamphetamine Use Disorder: A Meta-Analysis

**DOI:** 10.3389/fpsyt.2020.585852

**Published:** 2020-11-19

**Authors:** Li He, Yanhui Liao, Qiuxia Wu, Tieqiao Liu

**Affiliations:** ^1^Department of Psychaitry, National Clinical Research Center for Mental Disorders, The Second Xiangya Hospital of Central South University, Changsha, China; ^2^Department of Psychiatry, Sir Run Run Shaw Hospital, School of Medicine, Zhejiang University, Hangzhou, China

**Keywords:** BDNF Val66Met, polymorphism, methamphetamine, addiction, meta-analysis

## Abstract

**Background:** Several studies had examined the association between brain-derived neurotrophic factor (BDNF) Val66Met polymorphism and methamphetamine (METH) use disorder, whereas the results were conflicting. The aim of this study was to conduct a meta-analysis to achieve a pooled effect size of the association between BDNF Val66Met polymorphism and METH use disorder.

**Methods:** Literature searches were conducted in PubMed, EMBASE, and Cochrane Library up to July, 2020. All relevant studies on the relationship of BDNF Val66Met polymorphism and METH addiction were retrieved. Pooled odds ratios (ORs) with 95% confidence intervals (95% CIs) were calculated in the dominant, recessive, co-dominant, and allele model to appraise the association.

**Results:** Seven case–control studies with a total of 2,204 subjects (956 METH-dependent cases and 1,248 healthy controls) were included in this meta-analysis. The results showed a significant correlation between BDNF Val66Met polymorphism and METH dependence in overall population under different genetic models. However, subgroup analysis indicated that the association only existed in Han Chinese but not in other Asian populations.

**Conclusion:** Although the current data indicate that BDNF Val66Met polymorphism might be a potential genetic factor for METH use disorder, more researches are needed to prove its role in different populations.

## Introduction

Methamphetamine (METH) is highly abused throughout the world, which remains an extremely serious public health issue. Although efforts to investigate the biological processes of METH use disorder (i.e., abuse, dependence, and psychosis) have uncovered several potential pathogenic factors, there is still a lack of putative biomarkers to predict its susceptibility. Former family and twins studies indicated that the development of substance use disorders was closely connected with genetic factor (1–3). Kendler et al. (4) found a heritability estimate of 57% for stimulants abuse/dependence in males, while Mcgue et al. (5) found that the heritability of use and abuse of amphetamines was 16% in adolescents. Besides, many genetic animal models supported the effects of genetic factors on METH addiction (6, 7). However, to date, the key genetic variations that increase susceptibility to METH addiction have not been identified. Brain-derived neurotrophic factor (BDNF) belongs to the neurotrophin family, which is widely expressed in the adult brain and plays a key role in the facilitation of development, differentiation, and survival of midbrain dopaminergic neurons as well as the modulation of synaptic transmission. Many reports showed that BDNF was involved in the development of psychiatric and substance use disorders and had a potential role in the treatment of these disorders (8–11). Experiments on animal models showed that increasing BDNF expression could promote the survival and protection of dopaminergic neurons following METH administration (12, 13).

BDNF Val66Met (rs6265; G196A), an early found functional single-nucleotide polymorphism (SNP) in BDNF gene, resulted in a valine (Val)-to-methionine (Met) substitution in the prodomain. BDNF Val66Met not only affected the secretion of BDNF but also changed the structure of BDNF and affected its interaction with HAP1 and Sortilin1 (14–16). BDNF Val66Met was involved in the modulation of neuronal morphology and synaptic plasticity as well as the development of neuronal, psychiatric, and drug use disorders (17–23). Researches suggested that BDNF Val66Met polymorphism was a potential predisposing genetic factor for different substance use disorders, such as cigarette, alcohol, cocaine, heroin, and METH (24–28). Significant differences in BDNF Val66Met genotype distribution were found between METH addicts and controls, and higher 66Val allele frequencies were found in both Asian and Caucasian drug addiction cases (28, 29). Excessive use of METH can induce psychotic episodes. Greening et al. (30) found that METH differentially altered dopamine signaling markers between mice with different BDNF Val66Met genotypes (Val/Val vs. Met/Met), implicating involvement of BDNF in Meth-induced reprogramming of the mesolimbic proteome, while the research by Sim et al. (31) suggested that the BDNF Val66Met polymorphism might contribute to METH dependence and psychosis in the Chinese population but not in other Malaysian ethnicities.

As mentioned above, though more and more studies have found that METH addiction is associated with BDNF, there are still many contradictions about the association between Val66Met polymorphism and METH addiction. The reason for the conflicting results might be ethnic specificity, limited samples, participant heterogeneity, and insufficient statistical ability. Therefore, the meta-analysis aimed at synthetizing current studies on the association of BDNF Val66Met polymorphism and susceptibility to METH use disorder.

## Methods

### Literature Search

Any literature concerning the association between BDNF polymorphisms and METH addiction was sought by using the Medical Subject Headings terms “methamphetamine,” “polymorphism,” “variant,” “BDNF,” “brain-derived neurotropic factor,” “rs6265,” “rs6265 G>A,” “G rs6265A,” and “Val66Met” in databases (PubMed, EMBASE, and Cochrane Library) up to July, 2020. To find more appropriate studies, the references of the key studies were hand-searched. When detailed information for calculating effect size was lacking, we contacted the authors directly. The study was registered at PROSPERO (CRD42020193010).

### Inclusion and Exclusion Criteria

Case–control and cross-sectional studies were included irrespective of publication status or language. In this meta-analysis, all included studies met the following criteria: (a) studies focused on the association between BDNF Val66Met polymorphism and METH use disorder susceptibility, (b) patients were diagnosed using standard diagnostic criteria by a psychiatrist, (c) genotypes distribution of the control group should be in Hardy–Weinberg Equilibrium (HWE), and (d) there were sufficient data of the genotypes in the case and control group to compute the odds ratios (ORs) and 95% confidence intervals (CIs). The exclusion criteria were as follows: (a) METH-dependent subjects involving other substance use disorders, (b) publications with patients with other neuropsychiatric disorders, and (c) no genotypic data available.

### Quality Assessment

The methodological quality assessment of inclusion studies was performed with the Newcastle-Ottawa scale (NOS) (32). A “star system” has been used to assess data quality based on three aspects: the selection, comparability, and exposure or outcome of interest. Two independent researchers identified the literatures, and divergences between reviewers were resolved through discussion or with the assistance of a third researcher. The power of meta-analysis was estimated by STATA 12.0 software (Stata Corporation, College Station, Texas, USA). When the power of test was higher than 0.8, the result was considered acceptable.

### Data Extraction

Data extraction from an individual study included the following: first author, publication year, site of study, ethnicity, sample size, age, gender distribution, diagnosis criteria, genotyping method, HWE of cases and controls, and distribution of genotypes. Two researchers extracted the data independently, and disagreements were settled through discussion or with the assistance of a third researcher.

### Statistical Analysis

Statistical analysis was performed by STATA 12.0 software (Stata Corporation, College Station, Texas, USA). The relationship between BDNF Val66Met polymorphism and METH use disorder was appraised by pooled ORs and 95% CIs in dominant model (Met/Met + Val/Met vs. Val/Val), recessive model (Met/Met vs. Val/Val + Val/Met), co-dominant model (Met/Met vs. Val/Val; Val/Met vs. Val/Val), and allele model (Met vs. Val). Summary OR results and publication bias were evaluated by *Z* test and Egger's test, respectively. *P* < 0.05 was considered statistically significant. *I*^2^ and *Q* test statistics were employed to ascertain the heterogeneity among studies. When the heterogeneity was high (*I*^2^ > 50% or *P* < 0.1), a random-effect model was selected. Otherwise, the fixed effects model was chosen. Subgroup analyses were conducted according to ethnicity. Sensitivity analysis was performed with leave-one-out approach to examine the stability of analysis.

## Results

### Literature Search

**Figure 1:**
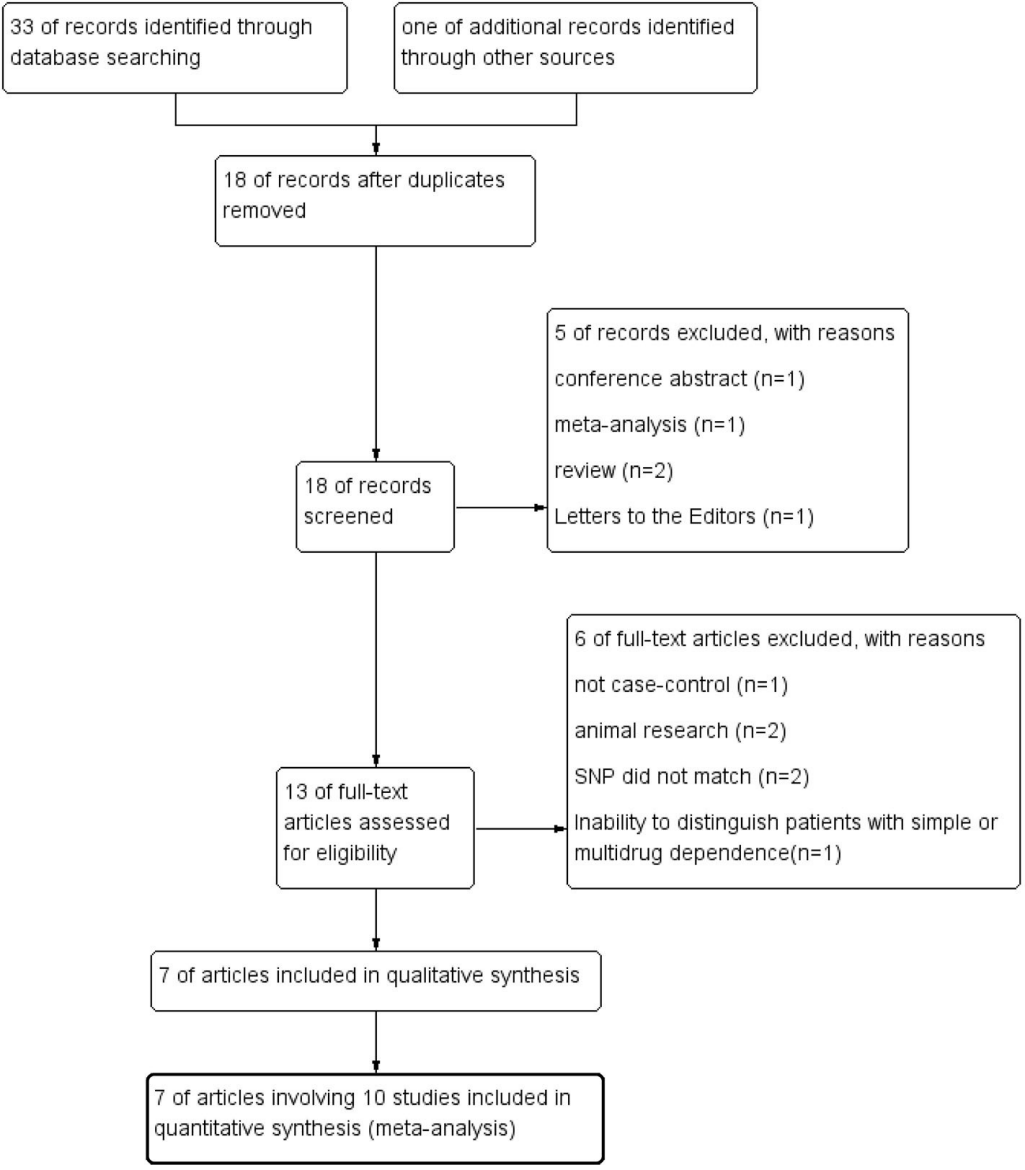
Flow diagram of literature retrieval and selection.

The process of systematic literature search and selection was displayed in Figure 1. We identified 33 articles through database searching and 1 additional article through reference lists searching. After excluding duplicates, 18 articles remained. Screening the titles and abstracts of these 18 articles resulted in 13 relevant studies. After the full-text review of the remaining 13 articles, 7 studies were qualified for final data analysis.

### Study Characteristics

Seven case–control studies (28, 29, 31, 33–36) with a total of 2,204 subjects (956 METH-dependent cases and 1,248 healthy controls) were included in this meta-analysis. Table 1 summarized the general characteristic of inclusion studies. All included studies were in accordance with HWE. The NOS scores of all studies were >6, indicating that they had good methodological quality (Supplementary Table 1). Except for one (33) employing ICD-10 criteria, all studies used DSM-IV criteria to identify METH-dependent subjects. One study from Malaysia were carried out in four different ethnic groups (31). After classification of this study into four subgroups, the number of studies was raised to 10.

**Table 1:** General characteristics of the included studies in the meta-analysis.

**References**	**Origin**	**Population**	**Sample size (case/control)**	**Sex Male (case/control)**	**Age (case/control) (mean ± SD)**	**Diagnosis**	**Genotyping**	**Genotypes distribution (case/control)**			**Allelic distribution (case/control)**		***p_**HWE**_* (case/control)**
								**Met/Met**	**Met/Val**	**Val/Val**	**Met**	**Val**	
Cheng et al. (28)	Taiwan	Han Chinese (Asian)	103/122	103/122	27.8 ± 6.8/29.8 ± 8.5	DSM-IV	PCR-RFLP	15/31	57/68	31/23	87/130	119/114	0.17/0.19
Itoh et al. (33)	Japan	Japanese (Asian)	189/202^a^	150/158	36.6 ± 11.9/37.2 ± 10.6	ICD-10	PCR-RFLP	23(8^a^)/25	96(27^a^)/107	70(21^a^)/70	43/157	69/247	0.25/0.10
Bousman et al. (29)	USA.	American (Caucasian)	117/76	117/76	36.6 ± 11.9/37.2 ± 10.6	DSM-IV	MassArray	2/2	29/29	86/45	33/33	201/119	0.80/0.29
Sim et al. (Malay) (31)	Malaysia	Malay (Asian)	59/51	59/51	31 ± 7.8/36 ± 10.6	DSM-IV	PCR-RFLP	6/10	33/25	20/16	45/45	73/57	0.15/0.97
Sim et al. (Chinese) (31)	Malaysia	Han Chinese (Asian)	24/45	24/45	40 ± 9.6/30 ± 9.4	DSM-IV	PCR-RFLP	2/12	12/27	10/6	16/51	32/39	0.54/0.14
Sim et al. (Kadazan-Dusun) (31)	Malaysia	Kadazan-Dusun (Asian)	50/30	50/30	29 ± 6.6/33 ± 13.1	DSM-IV	PCR-RFLP	9/6	28/18	13/6	46/30	54/30	0.37/0.27
Sim et al. (Bajau) (31)	Malaysia	Bajau (Asian)	53/28	53/28	28 ± 6.4/31 ± 16.7	DSM-IV	PCR-RFLP	14/6	26/14	13/8	54/26	52/30	0.89/0.98
Su et al. (34)	China	Han Chinese (Asian)	200/219	167/173	30.79 ± 7.99/33.68 ± 9.87	DSM-IV	MassArray	40/50	96/118	64/51	176/218	224/220	0.71/0.25
Iamjan et al. (35)	Thailand	Thai (Asian)	100/102	100/102	NA	DSM-IV	Real-time PCR	20/25	42/54	38/23	82/104	118/100	0.19/0.55
Su et al. (36)	China	Han Chinese (Asian)	194/378	160/149	31.46 ± 8.40/45.99 ± 12.96	DSM-IV	MassArray	39/90	93/189	62/94	171/369	217/377	0.70/0.79

a *Fifty six patients abuse methamphetamine only in their lifetime and 122 patients abuse some other drugs besides methamphetamine in the present or past; p_HWE_, p-value for Hardy–Weinberg equilibrium; NA, not available*.

Of the 10 different cohorts included in our study, 9 were from Asia (28, 31, 33–36). Two studies from China, one study from Taiwan, and one study from Malaysia were carried out in the Han Chinese population (28, 31, 34, 36). Cheng et al. (28) studied BDNF Val66Met polymorphism in both METH- and heroin-dependent patients, and only the former was included in the study. In the study by Itoh et al. (33), 56 patients abused MTEH only, while 122 patients abused other drugs besides METH. According to the predetermined inclusion criteria, the 56 subjects with METH dependence only were included in the data analysis. Four of the included (28, 29, 31, 35) studies were carried out in male only. Itoh et al. (33) analyzed the association of two BDNF gene SNPs with METH abuse in Japan. Bousman et al. (29) investigated association of six putative SNPs in six genes (AKT1, ARRB2, BDNF, COMT, GSTP1, and OPRM1) with METH abuse in Caucasian.

### Meta-Analysis

**Figure 2:**
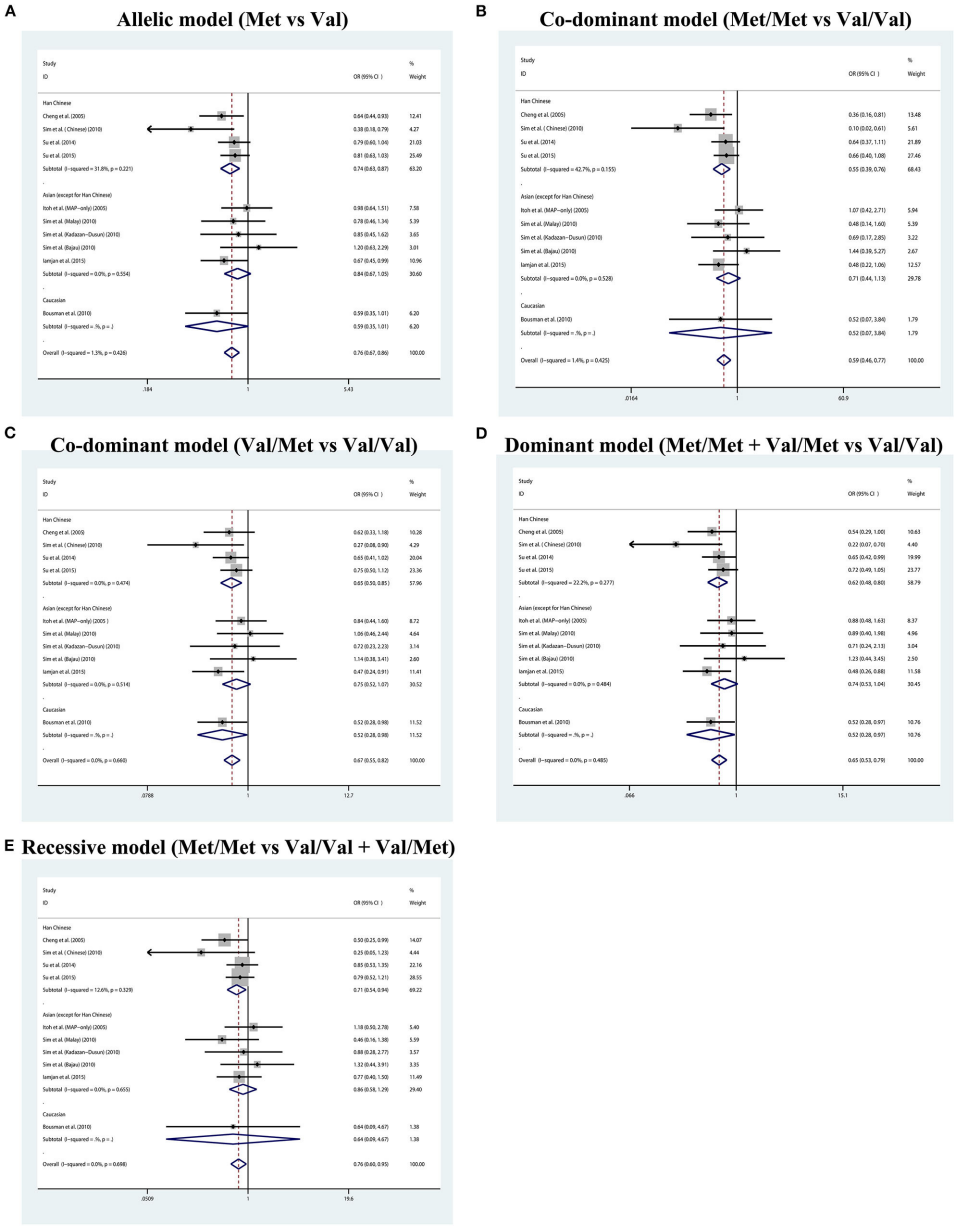
Forest plot for the association between BDNF Val66Met polymorphism and methamphetamine dependence susceptibility in five genetic models. **(A)** Allelic model (Met vs. Val). **(B)** Co-dominant model (Met/Met vs. Val/Val). **(C)** Co-dominant model (Val/Met vs. Val/Val). **(D)** Dominant model (Met/Met + Val/Met vs. Val/Val). **(E)** Recessive model (Met/Met vs. Val/Val + Val/Met).

**Figure 3:**
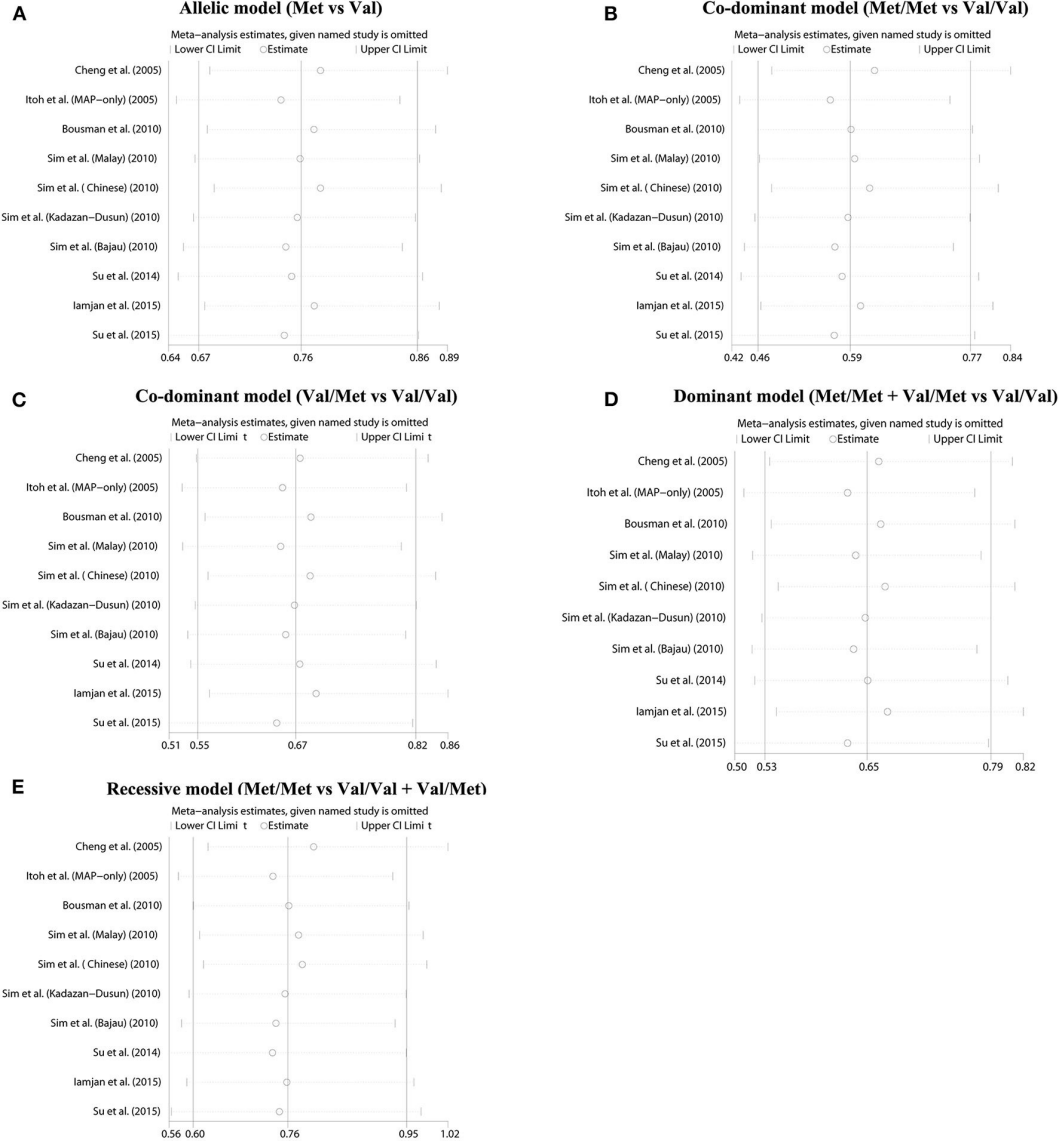
Sensitivity analysis results. **(A)** Allelic model (Met vs. Val). **(B)** Co-dominant model (Met/Met vs. Val/Val). **(C)** Co-dominant model (Val/Met vs. Val/Val). **(D)** Dominant model (Met/Met + Val/Met vs. Val/Val). **(E)** Recessive model (Met/Met vs. Val/Val + Val/Met).

The summary data of different genetic models indicated a significant association between BDNF Val66Met polymorphism and METH addiction in overall population, and no publication bias and significant heterogeneity were identified (Table 2 and Figure 2). Except for the recessive model, the power of meta-analyses was acceptable (Table 2). Figure 3 displayed the results of sensitivity analysis for different genetic models in the overall population. When the study by Cheng et al. was removed, the pooled OR changed a lot (from OR = 0.76, 95% CI: 0.60–0.95 to OR = 0.80, 95% CI: 0.63–1.02, Figure 3E) under the recessive model. While for the dominant, co-dominant, and allele model, the overall effect was not reversed after the deletion of study by Cheng et al. (Figures 3A–D). Therefore, this study was retained.

**Table 2:** The results of the meta-analyses under different genetic models for all studies.

**Genetic model**	**Comparison**	**Association**			**Heterogeneity**		**Publication bias^**a**^**	**Power of test**
		**OR**	**95% CI**	***P***	***I*^**2**^ (%)**	***P***		
Dominant model	Met/Met + Val/Met vs. Val/Val	0.65	0.53–0.79	<0.01	0.00	0.49	0.70	0.999
Recessive model	Met/Met vs. Val/Val + Val/Met	0.76	0.60–0.95	0.02	0.00	0.70	0.48	0.728
Co-dominant model	Met/Met vs. Val/Val	0.59	0.46–0.77	<0.01	1.40	0.43	0.55	0.990
	Val/Met vs. Val/Val	0.67	0.55–0.82	<0.01	0.00	0.66	0.80	0.991
Allele model	Met vs. Val	0.76	0.67–0.86	<0.01	1.30	0.43	0.59	0.998

a *P-value for Egger's test*.

Considering the conflicting results between different populations, the further subgroup analysis by ethnicity was employed. Four studies of Han Chinese origin and five studies of East and Southeast Asian origin (except for Han Chinese) were enrolled in the subgroup analysis (Table 3 and Figure 2). Due to insufficient data, we were unable to perform a subgroup analysis of Caucasian population, but we listed a study of Caucasian origin as a reference in Table 3 and Figure 2. In the subgroup analyses, we found a statistically significant association between BDNF Val66Met polymorphism and METH addiction in Han Chinese, but not in other Asian populations. Heterogeneities were increased in the Han Chinese group under different genetic models.

**Table 3:** The results of subgroup meta-analyses under different genetic models.

**Subgroup**	**Genetic model**	**Comparison**	**Association**			**Heterogeneity**	
			**OR**	**95% CI**	***P***	***I*^**2**^ (%)**	***P***
Asian (Han Chinese)	Dominant model	Met/Met + Val/Met vs. Val/Val	0.62	0.48–0.80	** <0.01**	22.20	0.28
	Recessive model	Met/Met vs. Val/Val + Val/Met	0.72	0.54–0.95	**0.02**	12.60	0.33
	Co-dominant model	Met/Met vs. Val/Val	0.55	0.39–0.76	** <0.01**	42.70	0.16
		Val/Met vs. Val/Val	0.66	0.50–0.86	** <0.01**	0.00	0.47
	Allele model	Met vs. Val	0.74	0.63–0.87	** <0.01**	31.80	0.22
Asian (except for Han Chinese)	Dominant model	Met/Met + Val/Met vs. Val/Val	0.74	0.53–1.04	0.08	0.00	0.48
	Recessive model	Met/Met vs. Val/Val + Val/Met	0.86	0.58–1.29	0.47	0.00	0.66
	Co-dominant model	Met/Met vs. Val/Val	0.71	0.44–1.13	0.15	0.00	0.53
		Val/Met vs. Val/Val	0.75	0.52–1.07	0.11	0.00	0.51
	Allele model	Met vs. Val	0.84	0.67–1.05	0.12	0.00	0.55
Caucasian^a^	Dominant model	Met/Met + Val/Met vs. Val/Val	0.52	0.28–0.97	**0.04**	/	/
	Recessive model	Met/Met vs. Val/Val + Val/Met	0.64	0.09–4.67	0.66	/	/
	Co-dominant model	Met/Met vs. Val/Val	0.52	0.07–3.84	0.52	/	/
		Val/Met vs. Val/Val	0.52	0.28–0.98	**0.04**	/	/
	Allele model	Met vs. Val	0.59	0.35–1.01	0.05	/	/

a *Only one study was included*.

*The bold values indicate that the p-value is less than 0.05*.

## Discussion

BDNF Val66Met, a non-synonymous SNP of BDNF gene, is related to a variety of neuropsychiatric diseases, such as Parkinson's disease, Alzheimer's disease, schizophrenia, and posttraumatic stress disorder (19–21, 37). Egan et al. (14) firstly examined the effects of BDNF Val66Met polymorphism and demonstrated a role for the variants in memory and hippocampal function. Subsequently, Greening et al. (30) found that BDNF Val66Met involved in METH-induced reprogramming of the mesolimbic proteome. The reward system of the midbrain and memory function of the hippocampus plays an important role in the formation of METH addiction. Therefore, due to the role of BDNF Val66Met in these processes, its relationship with METH addiction has attracted more and more attention. Many studies have examined the association between BDNF Val66Met polymorphism and METH abuse, but the results of these studies were not entirely consistent with each other. In this meta-analysis, we found that there was an association between BDNF Val66Met polymorphism and METH addiction in the overall population, suggesting that BDNF Val66Met polymorphism might be a potential marker for METH-dependent susceptibility. METH is a psychostimulant and has obvious neurotoxicity to dopamine neurons. In the animal METH-treated model, increased BDNF resulted in preservation of corpus striatal dopamine levels, indicating that the BDNF expression had a certain effect on antagonizing the nerve damage induced by METH (38). BDNF expression was increased in METH-dependent patients, and the BDNF Val66Met genotypes can affect the secretion of BDNF (36, 39). Therefore, the effects of BDNF Val66Met polymorphism on BDNF expression might be relevant to METH addiction.

As the use of METH penetrated the geographic regions of different ethnicities in the world, racial differences among METH abusers were observed (40). In the subgroup meta-analysis by ethnicity, we found a significant association between BDNF Val66Met polymorphism and METH addiction in Han Chinese, but not in other Asian populations. This result was different from that of Haerian et al., who found that the BDNF Val66Met polymorphism may be a risk factor for addiction to METH in a South Asian population (17). The difference might result from the differences in the included trials and subgroup analysis. In the study by Haerian et al., five inclusion studies investigated the association between BDNF Val66Met polymorphism and METH addiction, and one of them recruited patients with simple or multidrug dependence. In this meta-analysis, we only included studies that explored the relationship between simple METH addiction and BDNF Val66Met polymorphism. Therefore, seven trials were included in this meta-analysis, including four trials the same as the inclusion studies by Haerian et al. and three additional trials. Haerian et al. conducted subgroup analysis only according to Asian and Caucasian ethnicity. Our study further separated Han Chinese from Asian ethnicity; for many studies, it was found that the 66Val allele frequency in Han Chinese was significantly different from the controls and the weight of Han Chinese in the overall analysis was relatively high. This meta-analysis found that BDNF Val66Met might be a genetic factor for METH use disorder among the Han Chinese population. The allele frequency of BDNF Val66Met varied between different geographical areas. The frequency of 66Val allele in East Asians is 0.510, while that in South Asians is 0.807 (41). The distribution of BDNF Val66Met genotypes was also different among the inclusion studies, implying that there might be differences in susceptibility to METH among people with diverse genetic backgrounds. In the subgroup analysis, heterogeneities were increased in the Han Chinese group. This heterogeneity might result from the participants' heterogeneity among the inclusion studies. Of the four studies carried out in Han Chinese, half (28, 31) only included METH-dependent male as research subjects. Research has reported the gender differences in socio-demographic and clinical characteristics of METH abusers (42). Furthermore, Heinzerling et al. (43) found that there was a significant interaction between sex and BDNF Val66Met among METH-dependent patients. Although the biological mechanism contributing to these differences is unclear, additional researches on the different biological basis of response to METH among male and female are warranted.

In summary, considering the race specificity, limited samples, participant heterogeneity, and insufficient statistical ability of the current studies on the association between BDNF Val66Met polymorphism and simple METH use disorder, we applied a meta-analysis to get a pooled effect size, illustrating the potential effect of BDNF Val66Met on METH addiction. Besides, in the subgroup analysis according to the ethnicity, we found that the effect of BDNF Val66Met on METH addiction had ethnically specific differences. However, there were still several limitations in this meta-analysis. First of all, the majority of the inclusion studies were conducted on men, so the relationship between BDNF Val66Met polymorphism and gender cannot be observed among METH abusers. Second, the limitation of sample size made a relative low power of the recessive model. Besides, too few Caucasian studies were carried out, and a stratified analysis by Caucasians could not be done under different genetic models because of the insufficient frequency data. Finally, this study only focuses on the association between BDNF Val66Met polymorphism and METH-dependent susceptibility without considering gene–gene or gene–environment interactions. Therefore, this conclusion still needs to be supported by more researches carried out in different populations, and more researches are needed to be conducted.

## Conclusions

This is the first meta-analysis to study the relationship between simple METH addiction and BDNF Val66Met polymorphism. In this meta-analysis, we found that the expression of BDNF 66Met in METH-dependent patients was lower than that in the controls, indicating that BDNF 66Met might be a protective factor for METH addiction. Besides, in the subgroup analysis, we found that BDNF 66Met was differentially expressed only in the Han population, suggesting that the protective effect of BDNF 66Met might be ethnic-specific. The mechanism of BDNF Val66Met gene polymorphism on METH addiction in the Han population remains to be further studied. The current studies on association between METH addiction and BDNF Val66Met polymorphism are mainly from Asia, while few are from other districts. Therefore, the current results might be modified with the increase of studies in other regions. Considering these limitations, large-sample studies with different ethnicities should be conducted to determine the role of the BDNF Val66Met polymorphism in different populations.

## Data Availability Statement

The original contributions presented in the study are included in the article/Supplementary Material, further inquiries can be directed to the corresponding author/s.

## Author Contributions

LH and TL: study design. LH, QW, and YL data collection, analysis, and interpretation. LH: drafting of the manuscript. TL and YL: critical revision of the manuscript. All authors approval of the final version for publication.

## Conflict of Interest

The authors declare that the research was conducted in the absence of any commercial or financial relationships that could be construed as a potential conflict of interest.
