# Supplementary material for: Association Between Brain-Derived Neurotrophic Factor Val66Met Polymorphism and Methamphetamine Use Disorder: A Meta-Analysis
Source: Front Psychiatry. 2020 Nov 19;11:585852. doi: 10.3389/fpsyt.2020.585852 (PMC7716815; doi:10.3389/fpsyt.2020.585852)
Supplement: Supplementary file 1 [file Table_1.DOCX]

Supplementary Table 1. Quality assessment of included studies by Newcastle-Ottawa Scale

| Item/Study | Cheng et al. | Itoh et al. | Bousman et al. | Sim et al. | Su et al. | Iamjan et al. | Su et al. |
| --- | --- | --- | --- | --- | --- | --- | --- |
| Adequate definition of cases | ★ | ★ | ★ | ★ | ★ | ★ | ★ |
| Representativeness of cases | ☆ | ☆ | ☆ | ☆ | ☆ | ☆ | ☆ |
| Selection of control subjects | ★ | ★ | ★ | ★ | ★ | ★ | ★ |
| Definition of control subjects | ★ | ★ | ★ | ★ | ★ | ★ | ★ |
| Control for important factor or additional factor | ★ | ★ | ★★ | ★ | ★ | ★ | ★ |
| Exposure assessment | ★ | ★ | ★ | ★ | ★ | ★ | ★ |
| Same method of ascertainment for all subjects | ★ | ★ | ★ | ★ | ★ | ★ | ★ |
| Non-response rate | ★ | ★ | ★ | ★ | ★ | ★ | ★ |

Notes: ★, star given; ☆, star not given; the definition/explanation of each column of the Newcastle-Ottawa Scale is available from http://www.ohri.ca/programs/clinical_epidemiology/oxford.asp.
